# Proteomic analysis of mismatch repair-mediated alkylating agent-induced DNA damage response

**DOI:** 10.1186/2045-3701-3-37

**Published:** 2013-09-19

**Authors:** Xi Chen, Yong Zhao, Guo-Min Li, Lin Guo

**Affiliations:** 1 State Key Laboratory of Virology, College of Life Sciences, Wuhan University, Wuhan, P. R. China; 2 Graduate Center for Toxicology and Markey Cancer Center, University of Kentucky Medical Center, Lexington, KY, USA; 3 Wuhan Institute of Biotechnology, Wuhan, P. R. China

**Keywords:** MNNG, DNA damage response, MSH6, SILAC-based quantitative mass spectrometry

## Abstract

**Background:**

Mediating DNA damage-induced apoptosis is an important genome-maintenance function of the mismatch repair (MMR) system. Defects in MMR not only cause carcinogenesis, but also render cancer cells highly resistant to chemotherapeutics, including alkylating agents. To understand the mechanisms of MMR-mediated apoptosis and MMR-deficiency-caused drug resistance, we analyze a model alkylating agent (*N*-methyl-*N*’-nitro-*N*-nitrosoguanidine, MNNG)-induced changes in protein phosphorylation and abundance in two cell lines, the MMR-proficient TK6 and its derivative MMR-deficient MT1.

**Results:**

Under an experimental condition that MNNG-induced apoptosis was only observed in MutSα-proficient (TK6), but not in MutSα-deficient (MT1) cells, quantitative analysis of the proteomic data revealed differential expression and phosphorylation of numerous individual proteins and clusters of protein kinase substrates, as well differential activation of response pathways/networks in MNNG-treated TK6 and MT1 cells. Many alterations in TK6 cells are in favor of turning on the apoptotic machinery, while many of those in MT1 cells are to promote cell proliferation and anti-apoptosis.

**Conclusions:**

Our work provides novel molecular insights into the mechanism of MMR-mediated DNA damage-induced apoptosis.

## Background

DNA mismatch repair (MMR) ensures genetic stability by correcting biosynthetic errors, suppressing non-homologous recombination, and mediating DNA damage-induced apoptosis [1-5]. While the first two functions of the MMR system prevent mismatch-derived mutations, the apoptotic function of the system is to initiate programmed cell death in cells with mutagenic and carcinogenic DNA lesions. The latter function has been recognized as an important factor in cancer chemotherapy, because tumor cells defective in MMR are highly resistant to chemotherapeutic drugs, including alkylating agents [6].

A key player in mammalian MMR is the major mismatch recognition protein MutSα, which is composed of MSH2 and MSH6 subunits. In a concerted action with other essential MMR factors that include MutLα, proliferating cell nuclear antigen (PCNA), replication protein A (RPA), exonuclease I, and DNA polymerases, MutSα targets the newly synthesized DNA strand for mismatch removal [2,7]. Previous studies have revealed that besides mismatch recognition, MutSα also recognizes base pairs that contain a variety of modified or damaged bases, including alkylating adduct *O*^6^-methylguanine (*O*^6^-MeG) [6,8,9]. It has been suggested that recognition of the *O*^6^-MeG:T or *O*^6^-MeG:C pair by MutSα possibly in complex with MutLα, activates the ATR/ATM signaling network to induce apoptosis, via either the futile cycle mechanism or the direct interaction and signaling mechanism [1,2,6,8,10]. Many downstream targets, such as p53, Chk1, Chk2, CDC25A, and SMC1, have been shown to play important roles in the MMR-dependent alkylation-induced apoptosis [8,10-12]. However, the molecular basis of the process, particularly how the DNA damage signal in the nucleus is transmitted to the cytoplasm/mitochondria to initiate apoptosis is unknown.

Using quantitative global proteomics and phosphoproteomics approaches, we have investigated in this study a pair of MMR-proficient and -deficient human lymphoblastoid cell lines, TK6 and MT1, for their responses to the treatment of *N*-methyl-*N*′-nitro-*N*-nitrosoguanidine (MNNG), a widely used model chemical for alkylating agent-induced mutagenesis, carcinogenesis, and cell killing [13,14]. TK6 and MT1 are two closely-related cell lines with dramatically different susceptibility to MNNG-induced DNA damage. Both cells do not express methylguanine methyl transferase (MGMT), a direct reversal repair enzyme that protects cells from MMNG-induced cytotoxicity by transferring the methyl group from *O*^6^-MeG to an internal cysteine of the enzyme, restoring Watson-Crick base pairing at the site of the damage [15]. MT1 cells were derived from MMR-proficient TK6 cells by mutagenesis [16], and are defective in the MSH6 subunit of MutSα, thereby defective in MMR [17,18]. As a result, MT1 cells are 500-times more resistant to killing by MNNG cytotoxicity than their parental TK6 cells. The differential response of TK6 and MT1 cells to MNNG underscores the critical role played by MMR in alkylating agent-induced apoptosis/DNA damage response (DDR) and makes these cells the ideal subject for this study.

We demonstrate here that TK6 and MT1 cells display significant differences in protein phosphorylation species, stoichiometry and abundance, and in activation of protein kinases and signaling networks in response to MNNG treatment. Generally, MNNG-induced alterations in TK6 cells promote apoptosis, but changes in MT1 cells stimulate cell proliferation. Our data provide significant new insights into the mechanism of MMR-mediated DNA damage-induced apoptosis.

## Results and discussion

### Cell cycle progression in MNNG-treated TK6 and MT1 cells

**Figure 1:**
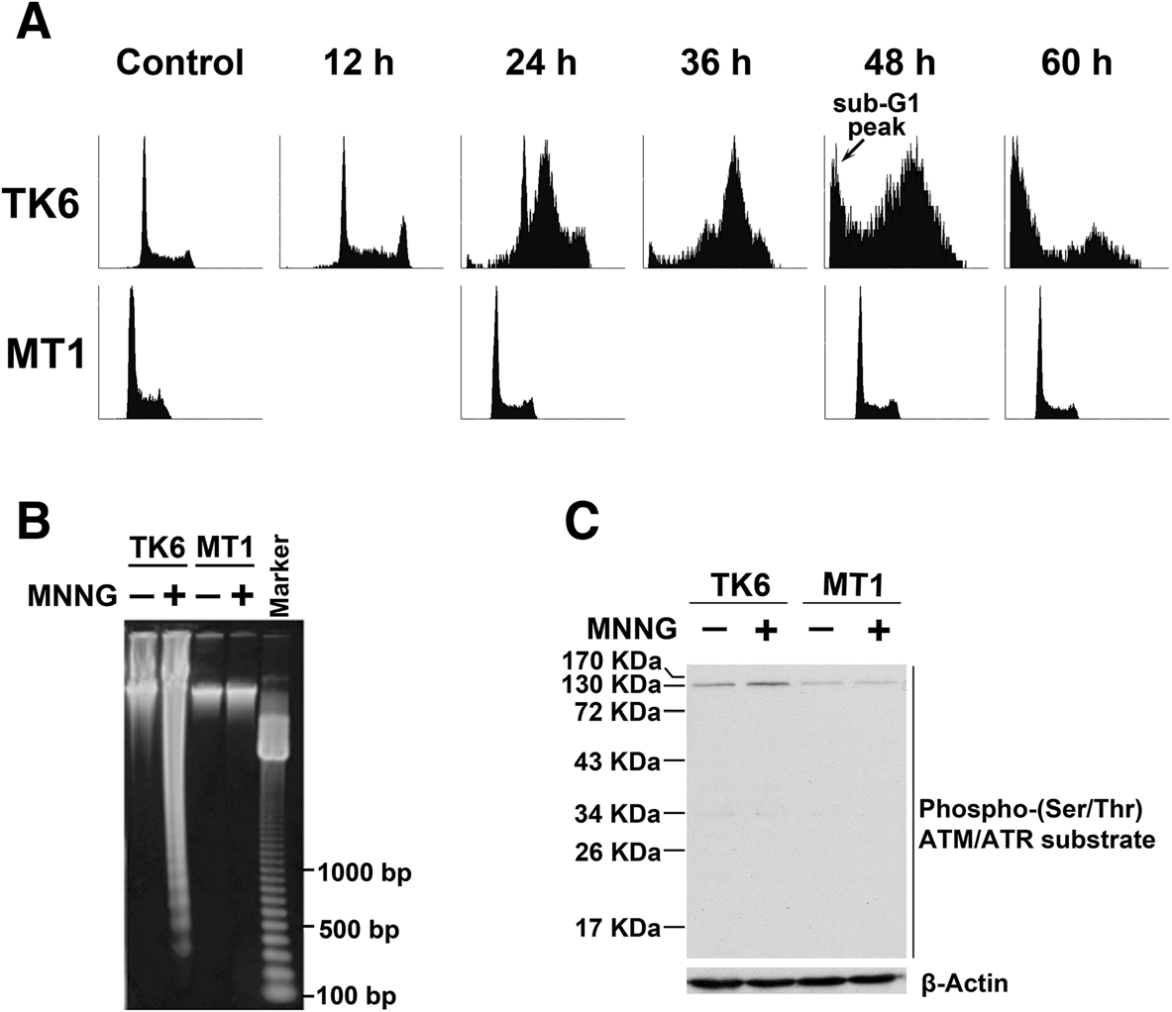
**Differential cellular responses to MNNG treatment in TK6 and MT1. (A)** Flow cytometric analysis of cell cycle progression. TK6 and MT1 cells were treated with 0.5 μM MNNG for 4 h, continuously cultured in fresh media without MNNG, and harvested for flow cytometry analysis at the indicated time points after MNNG treatment. **(B)** DNA fragmentation analysis. TK6 and MT1 cells were treated with 0.5 μM MNNG for 4 h, continuously cultured in fresh media without MNNG for 72 h, harvested and extracted for genome to analyze with DNA fragmentation experiment. **(C)** Detection of phosphorylation level of ATR/ATM kinase substrates at 4 h time point after 0.5 μM MNNG treatment. TK6 and MT1 cells were incubated in the presence or absence of 0.5 μM MNNG for 4 h. Whole cell lysates were analyzed by western blotting with an anti-phospho-(Ser/Thr) ATM/ATR substrate antibody. This antibody preferentially detects endogenous levels of proteins containing the ATM/ATR substrate motif. Equivalent gel loading was confirmed by probing with an antibody against β-Actin.

The goal of this study was to analyze MNNG-induced changes in the proteome and phosphoproteome of the MMR-proficient TK6 cell line and its *MSH6*-deficient derivative, MT1 [17,18]. To confirm their susceptibility to alkylating agent-induced apoptosis, TK6 and MT1 cells were exposed to 0.5 μM MNNG and cell cycle progression was analyzed by flow cytometry [19]. The results showed a higher fraction of TK6 cells in S phase 24 h after MNNG treatment, and a time-dependent increase in the fraction of cells in sub-G1, indicating induction of an S-phase checkpoint and apoptosis, respectively (Figure 1A). In contrast, exposure to the same concentration of MNNG had no effect on cell cycle distribution of MT1 cells (Figure 1A). DNA fragmentation analysis showed distinct DNA ladders in MNNG-treated TK6 cells, but not in MNNG-treated MT1 cells (Figure 1B). These data confirm that TK6 cells are highly susceptible and MT1 cell are highly resistant to MNNG-induced cell cycle arrest and apoptosis. In consistent with previous studies in HeLa and other human cells [8,11,12,20], a 4-hour exposure to 0.5 μM MNNG resulted in increased (~2.5-fold) phosphorylation of an ATR/ATM substrate with a molecular weight ~130 Kd in TK6 cells, but no change was observed in MT1 cells (Figure 1C), suggesting that activation of ATR/ATM signaling is dependent on a functional MMR system.

### Overview of changes in total and nuclear proteomes of MNNG-treated TK6 and MT1 cells

**Figure 2:**
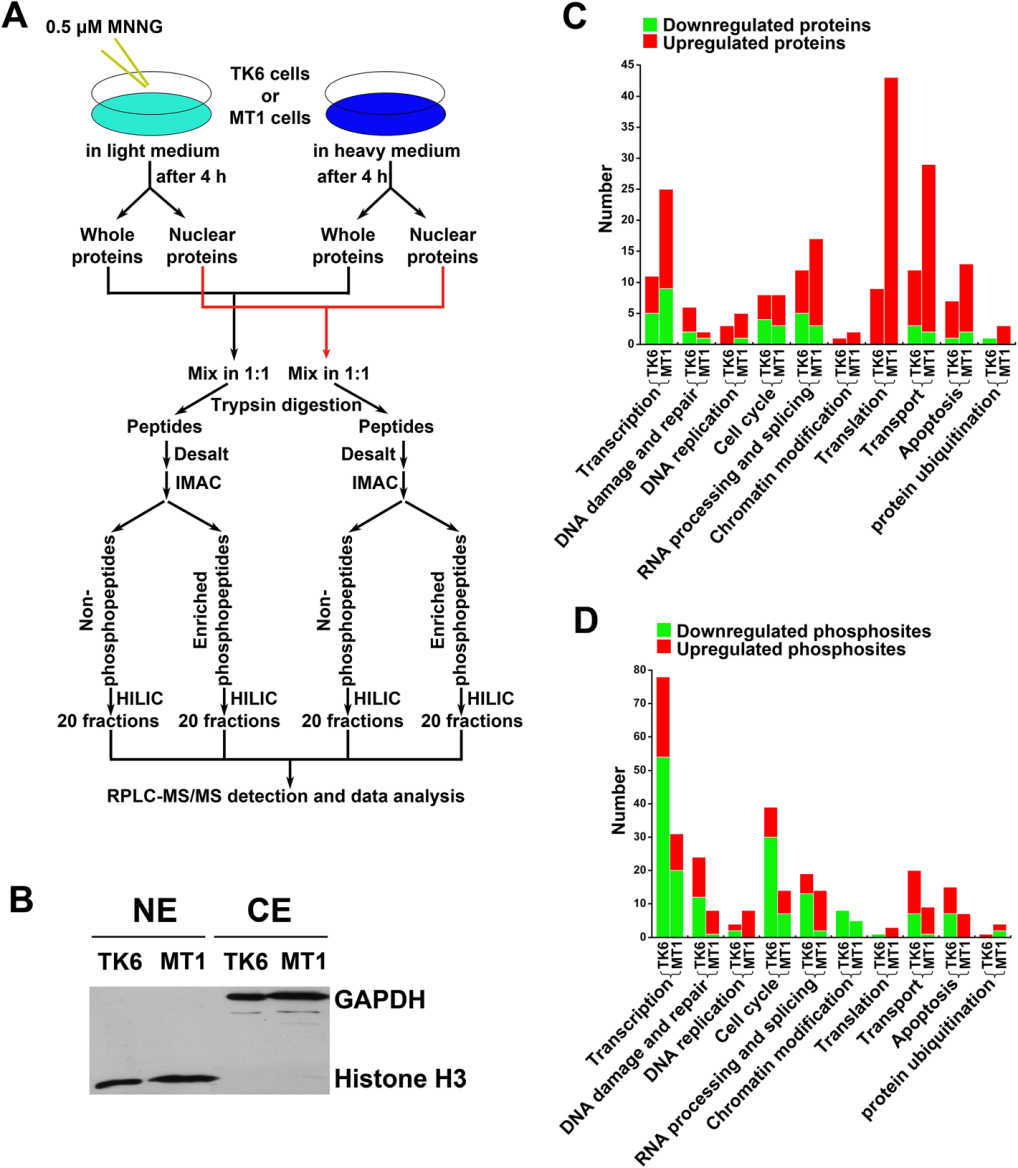
**Experimental strategy and overview of changed nuclear proteins in TK6 and MT1. (A)** Strategy for the identification and quantification of phosphorylation stoichiometry and protein abundance in TK6 and MT1 at 4 h after 0.5 μM MNNG treatment. **(B)** Validation of nuclear-extract’s purity using antibodies of GAPDH and Histone H3. **(C)** Functional distribution of changed proteins in TK6 and MT1. All the up- or down-regulated proteins from MNNG-treated TK6 and MT1 nuclear proteome were analyzed through their biological process distribution based on Gene Ontology (GO) annotation. **(D)** Functional distribution of changed phosphosites in TK6 and MT1. All the up- or down-regulated phosphosites from MNNG-treated TK6 and MT1 nuclear proteome were analyzed through their biological process distribution based on Gene Ontology (GO) annotation.

To evaluate changes in protein abundance and protein phosphorylation in MNNG-treated cells, we performed quantitative proteomics analyses. The strategy of these analyses is outlined in Figure 2A. The MNNG treatment groups were cultured in SILAC (Stable Isotope Labeling with Amino Acids in Cell Culture) light media, while control cells (no MNNG) were grown in heavy media. Following MNNG treatment, TK6 or MT1 cells were either lysed with RIPA (Radio Immunoprecipitation Assay) buffer for whole cell protein extracts or fractionated for preparation of nuclear extracts. Western blot, using antibodies against GAPDH and histone H3, was performed to confirm the purity of cytoplasmic and nuclear extracts, respectively (Figure 2B). Proteins were mixed in 1:1 (heavy:light) ratio and digested into peptides with trypsin. Peptides were separated into phosphopeptides and nonphosphopeptides by IMAC (Immobilized Metal Affinity Chromatography), then fractionated by HILIC (hydrophilic interaction chromatography) and analyzed by nano-RPLC-ESI-MS/MS (nano-reversed phase chromatography-electrospray ionization-MS/MS) analysis.

The number of identified and quantified unique proteins and phosphopeptides in the total and nuclear proteomes of each cell line is listed in Table 1. Detailed information regarding the identified proteins and phosphopeptides were listed in Additional file 1: Table S1 (proteins) and Additional file 2: Table S2 (phosphopeptides). Comparing data from the whole cell extract to those from the nuclear extract (Table 1), we found: 1) although the number of identified proteins in the whole cell extract were about 30% more than in the nuclear fraction (~1,700 proteins vs ~1,100 proteins), the number of identified phosphopeptides in both sets (~1,500) were comparable. This was mainly because the nuclear fractionation step made many lower-abundant nuclear proteins detectable, demonstrating that subcellular fractionation enhanced the sensitivity in our proteomic strategy. 2) More changes were documented in the nuclear proteome than in the total proteome. For example, the percentage of up- or down-regulated phosphopeptides in TK6 nuclear extract was ~18.3% (203 out of 1108 phosphopeptides quantified), while this ratio was only ~3.7% for the whole cell extract (28 of 766 phosphopeptides quantified). This further demonstrated the advantage and value of adding a subcellular fractionation step.

**Table 1:** Number of peptides and proteins identified and quantified in MNNG-treated cells

**Component**	**Whole cell extract**		**Nuclear extract**	
	**TK6**	**MT1**	**TK6**	**MT1**
Unique phosphopeptides	1438 (FDR^#^ = 1.9%)	1654 (FDR^#^ = 1.6%)	1594 (FDR^#^ = 2.7%)	1348 (FDR^#^ = 2.3%)
Unique proteins	1792 (FDR^#^ = 4.9%)	1765 (FDR^#^ = 4.5%)	1238 (FDR^#^ = 4.5%)	1102 (FDR^#^ = 4.8%)
Phosphopeptides quantified	766	925	1108	901
Proteins quantified	1153	1198	794	724
Down-regulated phosphopeptides^*^	13	13	127	37
Up-regulated phosphopeptides^*^	15	16	76	74
Down-regulated proteins^*^	30	20	23	16
Up-regulated proteins^*^	26	29	51	178

^#^The FDR (false-discovery rate) of phosphopeptides was calculated by searching query sequences against a randomized SwissProt human database with significance threshold *p* set to 0.05. The identified proteins were grouped by Scaffold (version 2_04_00) to eliminate redundancy and the FDR of proteins were also calculated by Scaffold software.

^*^The cutoff threshold was set at 1.5-fold increase or decrease, that is, a light-to-heavy ratio (L/H) ≥ 1.5 or ≤ 0.66.

Because significantly more up- or down- regulatory events were documented in the nuclear extract (Table 1), this directed our interest to the nuclear proteome, which is considered relevant to the MNNG-induced DDR. Therefore, we conducted detail analysis dissecting the key differences in the nuclear proteomes in MNNG-treated TK6 and MT1 cells.

### System-level differences between the nuclear proteomes in MNNG-treated TK6 and MT1 cells

To identify key differences between the nuclear proteomes in MNNG-treated TK6 and MT1 cells, we performed quantitative proteomics analyses and obtained nuclear proteomics profiles of the two cells (see Additional file 1: Table S1 and Additional file 2: Table S2). Systematical comparison of the nuclear proteomes allowed us to extract out all the up- or down-regulated proteins and phosphoproteins from MNNG-treated TK6 and MT1 cells. We further analyzed for the extracted data for their biological function distribution using Gene Ontology (GO) annotation, and discovered that more than 90% of the up- and down-regulated proteins and phosphoproteins could be categorized into ten GO categories. These GO categories include: Transcription, DNA damage and repair, DNA replication, Cell cycle, RNA processing and splicing, Chromatin modification and nucleosome assembly, Translation, Transport, Apoptosis and Protein ubiquitination. The number of up- and down-regulated proteins in MNNG-treated TK6 and MT1 cells that fall into the GO categories is shown in Figure 2C and 2D (details are listed in Additional file 3: Table S3), which reveals the following interesting phenomena.

First, we found that there were essentially more protein expression changes, particularly up-regulated proteins (see red bars in Figure 2C), in MT1 cells than in TK6 cells (194 in MT1 vs. 74 in TK6), but there were more protein phosphorylation level changes, especially down-regulated phosphosites (see green bars in Figure 2D) in TK6 cells than in MT1 cells (203 in TK6 vs. 111 in MT1). Interestingly, the changes in protein expression (Figure 2C) and protein phosphorylation (Figure 2D) were independent of each other. For example, although more changes in protein expression were observed for MT1 Transcription category (Figure 2C), more protein phosphorylation level changes were observed in TK6 cells (Figure 2D).

Second, our data show strong up-regulation of “Translation proteins” in MNNG-treated MT1 cells (43 proteins), while significantly fewer (9 proteins) “Translation proteins” were up-regulated in MNNG-treated TK6 cells (Figure 2C). If this correlates with more active translation in MNNG-treated MT1 cells than in MNNG-treated TK6 cells, then it could explain the higher total number of up-regulated nuclear proteins in these cells (Table 1). We hypothesize that the preferentially up-regulated “Translation proteins” in MNNG-treated MT1 cells may be responsible for tolerance to killing by MNNG toxicity. Indeed, some of the up-regulated proteins that we identified in MT1 cells, including EF2 (Elongation factor 2), EIF4G1 (Eukaryotic translation initiation factor 4 gamma 1), RPL5 (60S ribosomal protein L5) and RPL23A (60S ribosomal protein L23a) (as listed in Additional file 1: Table S1), have been previously implicated in anti-apoptosis, drug resistance and cell survival [21-25].

Third, from the protein phosphorylation perspective, in nearly all the GO categories outlined, we identified more alteration in MNNG-treated TK6 cells than in MNNG-treated MT1 cells (Figure 2D). This is especially evident in the GO categories of “Transcription” (78 vs. 31), “DNA damage and repair” (24 vs. 8), “Cell cycle” (39 vs. 14), and “Transport” (20 vs. 9) (Figure 2D). Because activities in protein phosphorylation and dephosphorylation in general correlated with activities in cellular signaling, we hypothesized what we observed may reflected a functional MMR pathway in TK6 directly or indirectly regulated DNA damage-related protein subnetworks during MNNG-stimulation, suggesting that functional MMR or functional MutSα might play an important role in modulating a series of biological processes, and ultimately leading to apoptosis in TK6 cells (Figure 1A and 1B).

### Unique proteomic changes in MNNG-treated TK6 and MT1 cells

**Figure 3:**
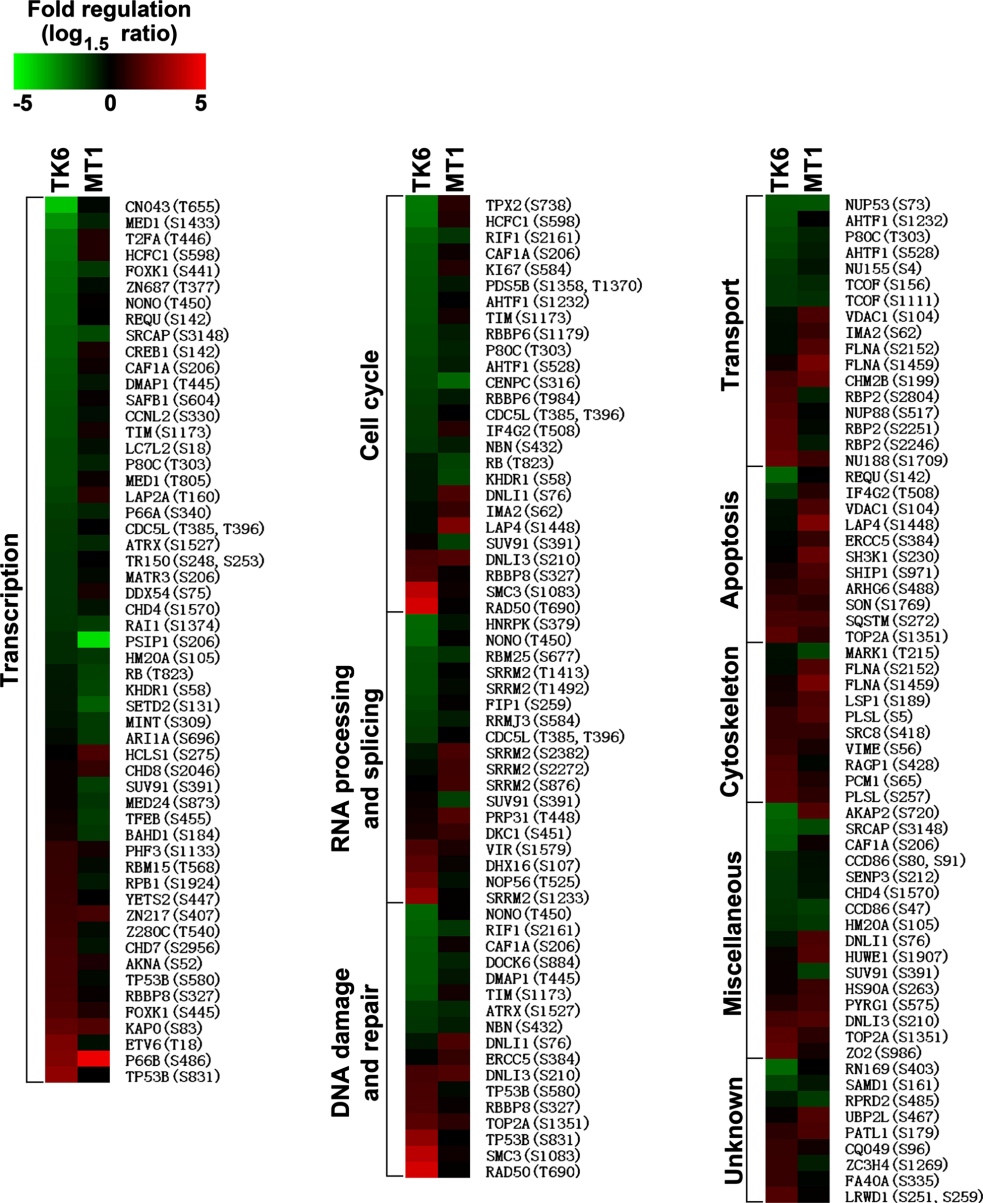
**Heat map of differential changes in protein phosphorylation between TK6 and MT1.** Phosphorylation sites with the following criteria were extracted for heat map: (1) same phosphosite was quantified in both MNNG-treated TK6 and MT1 nuclear fraction; (2) the phosphosite was up- or down-regulated in at least one cell type. The protein accession numbers and phosphosites were labeled, and all shown phosphoproteins were classified by their biological processes based on Gene Ontology (GO) annotation. Some phosphoproteins may exist in several different biological processes.

Because we conducted the TK6 and MT1 proteomics experiment independently (Figure 2A), some of the proteins were found in one experiment, but not in another, which is very common for mass spectrometry-based proteomics analysis [26]. In order to directly compare protein phosphorylation at individual protein level between TK6 and MT1, we compile a list of phosphorylation sites with the following criteria: 1) same phosphosite was quantified in both MNNG-treated TK6 and MT1 nuclear fraction; 2) the phosphosite was up- or down-regulated in at least one cell type. These phosphosites are listed in Additional file 4: Table S4, and a corresponding heat map is shown in Figure 3.

In the heat map (Figure 3), the color indicated the level of phosphorylation at individual protein phosphorylation sites, with the green and red colors being the lowest and highest levels, respectively (see color diagram at the top panel). In each of the GO categories shown in Figure 3, protein phosphorylation sites are listed according to their phosphorylation levels (from low to high) in TK6. From this analysis, we deduced that: 1) the direction of change in protein phosphorylation was rarely opposite in TK6 and MT1 cells; 2) a few phosphorylation were up- or down-regulated in both MNNG-treated cell lines; but 3) most phosphorylation were up- or down-regulated in one of the cell lines and unaffected by MNNG-treatment in the other cell line.

Among the 133 phosphorylation sites listed in Figure 3, only one phosphorylation site, namely Ser720 of AKAP2 shown opposite change in protein phosphorylation (see the first entry in the Unknown Miscellaneous category). AKAP2 protein can bind to regulatory subunit (RII) of protein kinase A [27] and may be involved in establishing polarity in signaling systems. The biological significance of AKAP2 protein in differential responses of TK6 and MT1 toward MNNG-treatment remains to be investigated.

**Figure 4:**
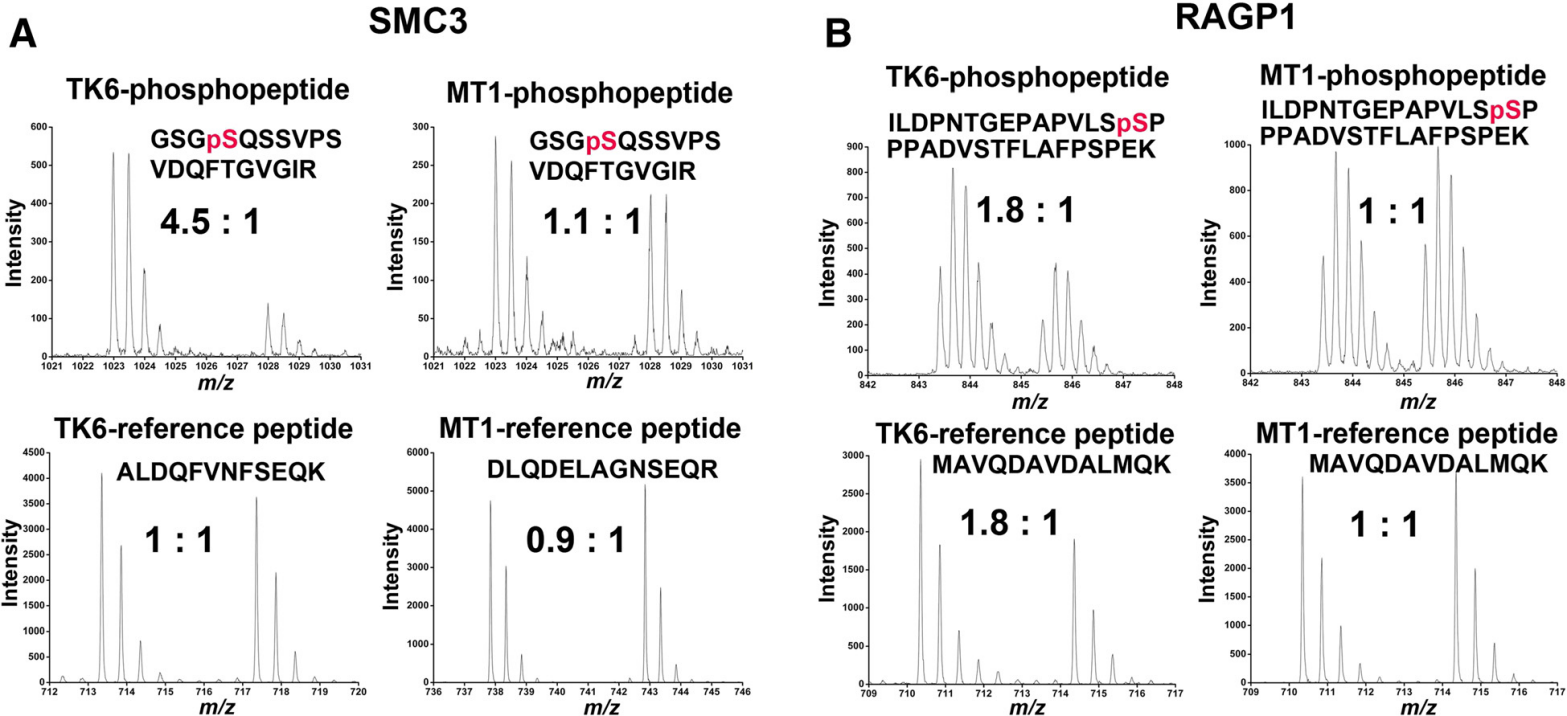
**Examples of peptide tandem mass spectra from differentially-regulated nuclear phosphoproteins of MNNG-treated TK6 and MT1 cells.** Peptide pairs were prepared using SILAC method, with the "light" labeling as MNNG treatment group. **(A)** Peptide pairs (GSGpSQSSVPSVDQFTGVGIR, ALDQFVNFSEQK, DLQDELAGNSEQR) from SMC3 protein, **(B)** Peptide pairs (ILDPNTGEPAPVLSpSPPPADVSTFLAFPSPEK, MAVQDAVDALMQK) from RAGP1 protein.

A number of protein phosphorylation sites were found to be up-regulated in MNNG-treated TK6 nuclear proteome, but not in MT1 (Figure 3). Many of these proteins, such as RAD50, RBBP8, SMC3, TOP2A and TP53B (see DNA damage and Repair category), were well known to participate in DDR network. Examples demonstrating these points are shown in Figure 4 and Additional file 5: Figure S1. To distinguish if a change in phosphorylation of a nuclear protein is due to increased post-translational modifications in the nucleus or translocation of the phosphorylated protein from the cytoplasm, we quantified a reference peptide that contains no phosphorylation sites from the same protein. Thus, if a protein of interest is elevated in nuclear extract as compared with the whole cell extract, it implies a nuclearcytoplasmic shuttling occurring; otherwise, the up-regulated phosphorylation result from the post-translational modifications in the nucleus. We found that phosphorylation of Ser1083 in SMC3 was differentially up-regulated in the nuclear proteome of MNNG-treated TK6 cells. This is because the level of the phosphorylated peptide in treated nuclear proteome is 4.5-fold higher than in untreated nuclear proteome (Figure 4A, upper left). However, the phosphorylation level of Ser1083 of SMC3 kept the same in treated and untreated MT1 cells (Figure 4A, upper right). Since the abundance of a non-phosphopeptide of SMC3 was unchanged (lower spectrum), the observed up-regulation of SMC3 phosphorylation in MNNG-treated TK6 proteome is a result of post-translational modifications by protein kinase(s) activated during the treatment. A similar result was also observed for Ser831 in TP53B and Ser1233 in SRRM2 (Additional file 5: Figure S1). All these three proteins are predicted substrates of ATM/ATR kinases (see below), whose role in DDR is well-established [28,29].

It is interesting to note that while we detected phosphorylation up-regulation of some important DDR factors in TK6 cells, we also identified more phosphorylation up-regulation of components involved in apoptosis in MNNG-tolerant MT1 cells than in MNNG-sensitive TK6 cells (see Figure 3, the GO category of “Apoptosis”). These proteins include anti-apoptotic ERCC5 (Ser384) and pro-apoptotic VDAC1 (Ser104), LAP4 (Ser1448), SH3K1 (Ser230), and SHIP1 (Ser971). This phenomenon appears to suggest that MNNG-induced phosphorylations have greatly altered the biological functions of these apoptosis-related proteins, i.e., inhibiting the pro-apoptotic activities of VDAC1, LAP4, SH3K1 and SHIP1, but enhancing the anti-apoptotic activity of ERCC5. However, further investigations are required to verify these possibilities.

### Activation of nucleocytoplasmic shuttling in TK6 cells upon exposure to MNNG

The comparative mass spectrometry analysis of phosphoprotein between nuclear extract and whole cell extract allowed us to identify an important phenomenon associated with MNNG treated TK6 cells, i.e., activation of nucleocytoplasmic transport. As shown in Figure 4B, phosphorylation of Ser428 in Ran GTPase-activating protein 1 (RanGAP1), a critical regulator of the small GTPase Ran (RAN)-dependent nucleoplasmic transport [30-32], was up-regulated 1.8 fold in the nuclear proteome of MNNG-treated TK6 cells, but the up-regulation was not detected in MNNG-treated MT1 cells (upper spectra). Because RanGAP1 is located primarily within the cytoplasm [33] and because Ser428 phosphorylation was observed previously in an MNNG-independent manner [34], we suspected that the up-regulated RanGAP1 level in the TK6 nucleus is likely due to transport of the protein from the cytoplasm. Indeed, we observed 1.8-fold up-regulation of a reference peptide of RanGAP1 in the TK6 nuclear extract (Figure 4B, lower spectrum), but not in the TK6 whole cell extract (data not shown). These results strongly suggest that the up-regulated nuclear RanGAP1 in MNNG-treated TK6 cells is a result of translocation of the protein from cytoplasm. Similarly, we detected nucleocytoplasmic shuttling of RAN, an essential player of the RAN-dependent nucleocytopasmic transport [30,31]. Since this phenomenon was only observed in MNNG-treated TK6 cells, the activation of the RAN/RanGAP1-dependent nucleocytoplasmic shuttling could be part of the MMR-mediated signaling network. Further studies are required to explore this possibility.

### A DDR protein interaction network in TK6 cells

When comparing MNNG-treated nuclear phosphoproteome in TK6 and MT1 cells, we noticed significantly more protein phosphorylation level changes in TK6 cells than in MT1 cells (203 in TK6 vs. 111 in MT1). Are these regulations on protein phosphorylation related to a DDR protein interaction network? To address this question, all phospho- and nonphospho-proteins identified in TK6 (as listed in Additional file 1: Table S1 and Additional file 2: Table S2) were queried for protein interactions using the program STRING (version 8.2) (http://string-db.org/) [35]. STRING integrates known and predicted interactions from multiple sources to generate a putative interaction network map for a given data set. A network of 1285 proteins (nodes) and 8427 connections (edges) were obtained (Additional file 6: Figure S2).

**Figure 5:**
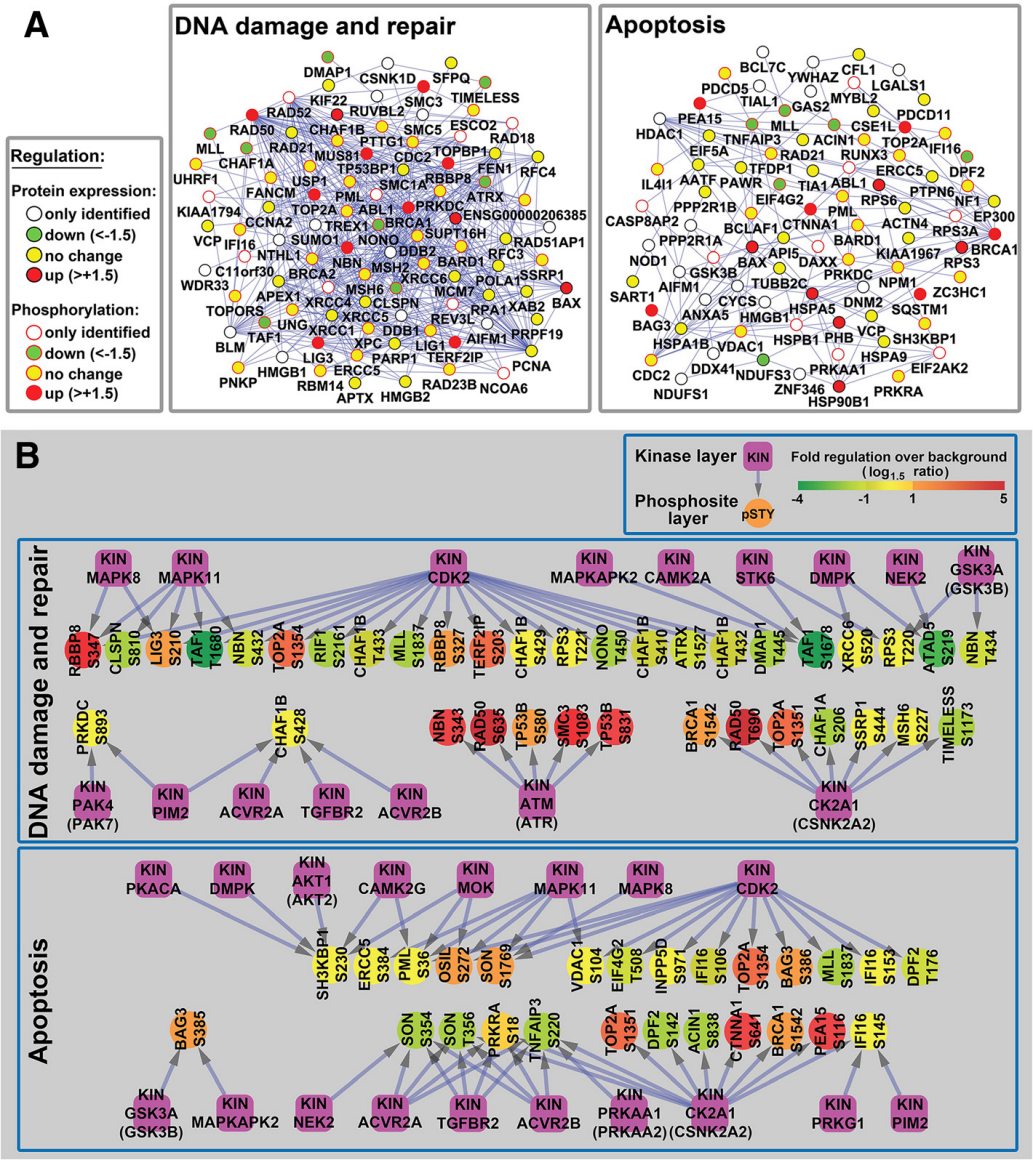
**Protein networks analysis in TK6 nuclear extract. (A)** Protein interaction Networks in TK6 nuclear extract. Protein phosphorylation and abundance data in TK6 nuclear were analyzed with the STRING database. A GO biological process analysis was performed to create two representative subgroups (“DNA damage and repair” and “Apoptosis”) If both protein expression and phosphorylation data were available, only information in phosphorylation was shown. **(B)** Kinase-substrate interaction networks in TK6 nuclear extract. NetworKIN algorithm was used to predict potential kinases for quantified phosphosites in “DNA damage and repair” and “Apoptosis” biological processes. The color of the phosphosites represented their alteration after MNNG treatment.

To enhance this data analysis, five subnetworks, comprised of proteins with shared GO terms/function, were examined in greater detail: the DNA damage and repair subnetwork included 90 proteins and 536 connections (Figure 5A, left panel), the Apoptosis subnetwork included 81 proteins and 155 connections (Figure 5A. right panel), the RNA processing and splicing subnetwork included 73 proteins and 435 connections (Additional file 6: Figure S2), the Cell cycle subnetwork included 89 proteins and 331 connections (Additional file 6: Figure S2), and 59 proteins and 102 connections were present in the Transcription subnetwork (Additional file 6: Figure S2). These five subnetworks included most of the TK6 MNNG-sensitive proteome subfraction, and included a highly interconnected group of TK6 proteins. Furthermore, for the proteins in these five subnetworks, MNNG-induced changes in phosphorylation status were more common than MNNG-induced changes in protein abundance. Thus, protein phosphorylation clearly mediates critical regulatory events during the early stages of MNNG-induced DDR in MMR-proficient TK6 cells.

ATM (ataxia telangiectasia mutated) and ATR (ATM and Rad3-related), members of the phosphoinositide 3-kinase related kinase (PIKK) family, are well-recognized master regulators of DDR. Together with other regulator and mediator proteins, ATM and ATR stimulate phosphorylation of Chk1 and Chk2, which in turn activate signaling cascades via a large number of downstream effector proteins, such as MCMs, RFC, TopBP1, Rad51 and FANCD2 [36]. Our proteomics data also showed increased expression or phosphorylation of BRCA1, NBN, RAD50, TP53BP1, SMC3, TOP2A, RBBP8, LIG3 and TERF2IP, all members of the DNA damage and repair subnetwork (Figure 5A). Previous studies suggested that NBN plays a role in the cellular response to MNNG [37], and that phosphorylation of SMC3, RAD50 and TP53BP1 increased in IR-treated cells [38]. Together, these data help validate our proteomics approach and analysis. However, a number of proteins with known roles in DDR, such as Chk1, Chk2, p53, RAD17, XRCC2, ERCC1 and MDC1, were not identified as DDR network components in this study. This may indicate that these low abundance proteins fell below the detection limit associated with the instrumentation and analytical procedures used in this study. On the other hand, we identified a number of novel putative DDR proteins events, which have not previously been linked to DDR (see below).

### Prediction of DDR protein kinase networks in MNNG-treated TK6 cells

Our quantitative phosphoproteomic analysis provided information regarding protein phosphorylation regulation upon MNNG-treatment. In order to use our data to explore protein kinases that may participate in DDR network, we used the NetworKIN algorithm to predict which kinases were responsible for MNNG-induced phosphorylation events within the DNA damage and repair, Apoptosis, Cell cycle and RNA processing and splicing subnetworks in TK6 cells (Figure 5B and Additional file 7: Figure S3).

We identified ATM or ATR as projected kinases for several proteins with up-regulation in phosphorylation. Because these protein substrates, NBN (Ser343), RAD50 (Ser635), TP53B (Ser580, Ser831) and SMC3 (Ser1083), are known players, and ATM/ATR are known master regulators in DDR, this finding further validated our phosphoproteomics approach in study cellular responses to MNNG treatment.

In addition, our analysis also project some other kinases, such as CDK2 (a cyclin-dependent protein kinase), Casein kinase II, and MAP kinase may be responsible for the phosphorylation of many protein substrates participating in “DNA damage and repair”, “Apoptosis”, “Cell cycle” and “RNA processing and splicing” in MNNG-treated TK6 cells (Figure 5B and Additional file 7: Figure S3), indicating that these kinases may also play important regulatory roles in MNNG-induced DDR.

Some proteins may be targeted by more than one protein kinase in MNNG-treated TK6 cells, suggesting that DDR involves coordinated regulatory activities and cross-talk between multiple kinases in TK6 cells. For example, S635 in RAD50, up-regulated 6.3-fold, is an ATM/ATR target, while T690 in RAD50, up-regulated 5.5-fold, is a target of Casein kinase II (Figure 5B).

Although unbiased phosphoproteomics analysis and system biology-based network investigation can provide insight into cellular signaling, in many cases, traditional molecular biology-based study, combined with functional assays are needed to identify and screen for the biological significance of predicted phosphorylation. Many protein phosphorylation sites we discovered are consistent with previous knowledge accumulated over the years using molecular biology method, giving credibility to our system biology level approach. For example, Ser343 phosphorylation in NBN (a ATM/ATR kinase substrate, Figure 5B) was identified previously as closely related with NBN-regulated apoptosis, and a mutation in the ATM/ATR phosphorylation site (Ser343A) leads to a severe inhibition of apoptosis [39]. The CDK2 kinase substrate RBBP8 (Ser327) (Figure 5B), a putative tumor suppressor, was recently shown to be involved in regulating the G2/M checkpoint through BRCA1-dependent ubiquitination in response to Ser327 phosphorylation [40] and to counteract Rb-mediated G1 restraint [41].

The large amount of information about regulations on protein phosphorylation generated from our study may provide new hints into mechanism of protein interaction. For example, Thr690 of RAD was identified as being up-regulated in MNNG-treated TK6 nuclear, with Casein Kinase II as its potential kinase (Figure 5B). RAD50 has a Cys-X-X-Cys motif of the zinc-hook structure located in the middle of the coiled-coil domain, which functions as a dimerization domain between two RAD50 arms [42]. The RAD50 hook is very important in forming a complex of MRE11/RAD50/NBN (MRN complex), which acts a double-strand break sensor for ATM and recruits ATM to broken DNA molecules and activates ATM to initiate phosphorylation-network-based apoptosis [42,43]. Interestingly, the Thr690 residue is located in the zinc-hook of RAD50. Therefore, it is speculated that the up-regulated phosphorylation on Thr690 site affects RAD50 dimerization, further influences the formation of MRN complex, leading to a series of downstream apoptosis-related protein interactions.

## Conclusions

**Figure 6:**
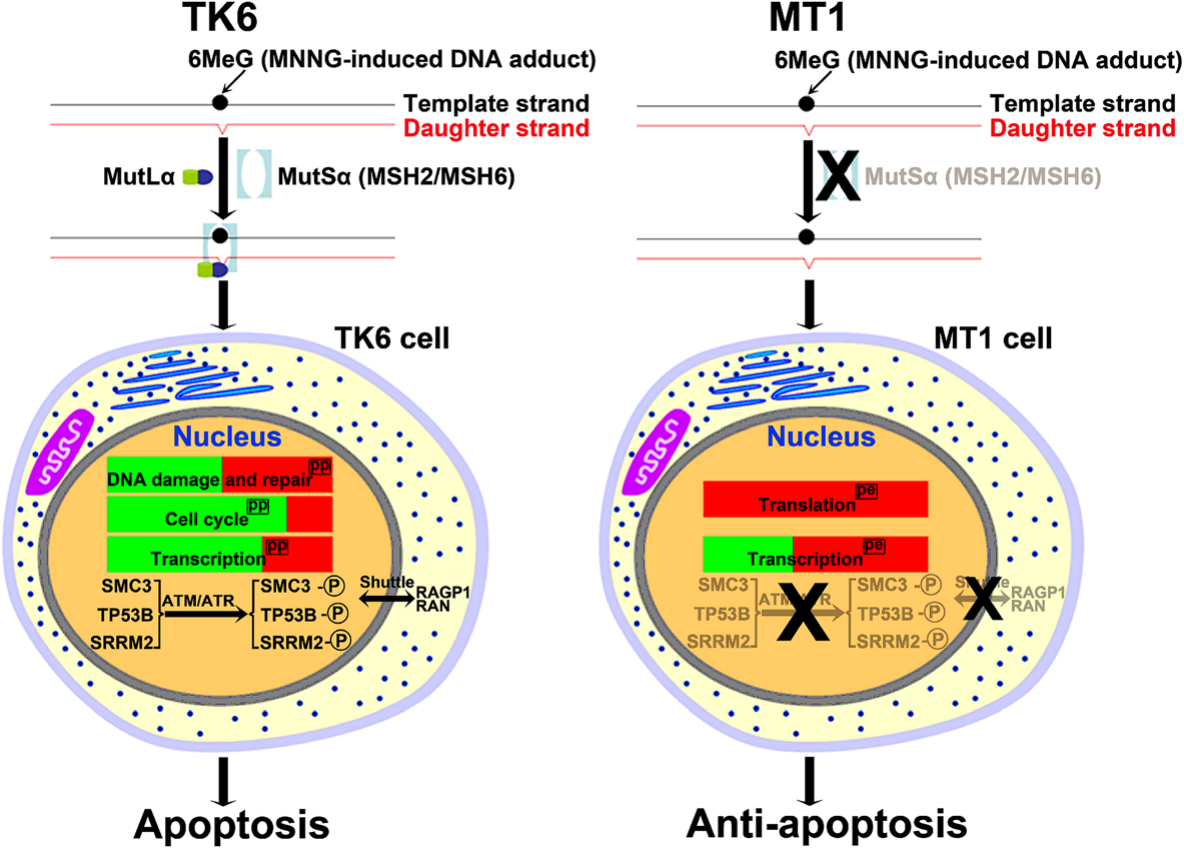
**Hypothetic mechanism of differential responses to MNNG treatment in TK6 and MT1.** The green or red bar represents the proportion of down- or up-regulated changes. (pp) represents protein-phosphorylation (pp) level change upon MNNG treatment. (pe) represents protein expression (pe) level change upon MNNG treatment.

TK6 and MT1 cells are two closely-related cell lines, mainly different in the existence of MSH6 [17,18]. However, MT1 cells are >500-fold more resistant to MNNG-induced apoptosis than TK6 cells [16]. Consistent with these genetic and phenotypic differences, the unbiased proteomics analysis in this study reveals significant differences in the nuclear proteome of TK6 and MT1 cells (Figures 2 and 3) during their early stage (4-hour) responses to MNNG treatment. Because MNNG not only can react with nucleic acid, it also can react with proteins [44], the differential responses in MNNG-treated TK6 and MT1 cells should be considered as the collective response of MNNG-nucleic acid and MNNG-protein interactions. A summary of main differential effects of MNNG on TK6 and MT1 proteome was illustrated in Figure 6. The key findings of this study include: 1) MNNG-induced phosphophorylation events are more frequent in TK6 than in MT1 cells (Table 1), suggesting that MSH6 plays an important role in initiating MNNG-induced DDR and apoptosis; 2) MNNG-induced changes (mostly up-regulation) of protein expression/abundance were more frequent in MT1 than in TK6 cells, especially for Translation proteins (Figure 2C), allowing MT1 to establish an anti-apoptotic response in the absence of MMR function; 3) ATM/ATR, CDK2, Casein kinase II and MAP kinases are predicted to play a role in DDR-associated phosphorylation events in MNNG-treated TK6 cells (Figure 5B and Additional file 7: Figure S3); 4) MNNG-treated TK6 cells may undergo RAN/RanGAP1-dependent nucleocytoplasmic transport of proteins, allowing shuttling of mRNAs and apoptotic stimuli between the nucleus and the cytoplasm, leading to initiation of apoptosis. However, it should be emphasized that the conclusions and the model are based on a single time-point “snap shot” of the TK6 and MT1 nuclear and total proteomes, and their confirmation requires additional thorough investigations.

## Methods

### Cell culture, flow cytometry and DNA fragmentation analysis

TK6 and MT1 cell lines were cultured in RPMI 1640 medium (GIBCO) supplemented with 10% heat-inactivated undialyzed fetal bovine serum (Hyclone), 100 U of penicillin and 100 μg/mL of streptomycin (Invitrogen) in a humidified incubator with 5% CO_2_ at 37°C.

For flow cytometry analysis, cells were treated or mock-treated with 0.5 μM MNNG and incubated for the indicated time periods. A total of 1.0 × 10^6^ cells were washed with ice-cold PBS and fixed in ice-cold 70% ethanol overnight. The cells were then treated with 100 μg/mL RNase A for 1 h at 37°C and stained with 50 μg/mL propidium iodide for 30 min at 4°C in the dark. Cell cycle analysis was performed using a BECKMAN COULTER EPICS XL flow cytometer and the EXPO32ADC software.

For DNA fragmentation analysis, cells were treated or mock-treated with 0.5 μM MNNG and incubated for the indicated time periods. A total of 5 × 10^5^ cells were washed with ice-cold PBS, centrifuged for 5 min at 400 × g, and resuspended in 40 μL cell lysis buffer (20 mM EDTA, 100 mM Tris, pH8.0 and 0.8% (w/v) SDS). RNase A (2 μL of 1 mg/mL, Fermentas) was added and incubated for 120 min at 37°C, then 10 μL Proteinase K (20 mg/mL, TaKaRa) was added and incubated for at least 90 min at 50°C. Sample was mixed with 10 × DNA loading buffer (TaKaRa), and loaded onto a 2% agarose gel. Electrophoresis was conducted at 2–4 V/cm for approximately 6 h. The DNA was stained with 1 μg/mL ethidium bromide (Sigma) in TAE buffer.

### SILAC labeling, MNNG treatment and western blot

Cells were washed twice with RPMI 1640 medium without Arg and Lys (Thermo Scientific Pierce) and reconstituted in RPMI 1640 medium containing either ^12^C_6_,^14^ N_4_ Arg, ^12^C_6_,^14^ N_2_ Lys (Sigma) and normal L-proline or ^13^C_6_,^15^ N_4_ Arg, ^13^C_6_,^15^ N_2_ Lys (Sigma) and normal L-proline. The media also contain 10% heat inactivated dialyzed fetal bovine serum (Thermo Scientific Pierce), 100 U of penicillin and 100 μg/mL of streptomycin. Cells were cultured in a humidified incubator (5% CO_2_) at 37°C for ~8 cell doublings. The concentration of Arg and Lys used in SILAC labeling of TK6 and MT1 cell lines was 0.398 mM and 0.798 mM respectively. Adding additional L-proline (200 mg/liter) should reduce arginine to proline conversion [45].

Cells were treated with or without 0.5 μM MNNG for 4 hours, and harvested by centrifugation at 400 g. Cell pellets were washed three times with cold PBS, and lysed for 30 min at 4°C in RIPA buffer (25 mM Tris–HCl pH 7.6, 150 mM NaCl, 1% NP-40, 1% sodium deoxycholate, 0.1% SDS) containing phosphatase inhibitor cocktail (PhosSTOP, Roche Applied Science) and protease inhibitor cocktail (COMPLETE, Roche Applied Science). After centrifugation for 15 minutes at 16000 g, the supernatant was collected and saved as whole cell protein extract.

For the immunoblotting, a small aliquot of proteins from MNNG-treated TK6 and MT1 cells were separated by SDS-PAGE, transferred electrophoretically onto a PVDF membrane (Amersham) and blocked for 1 h with TBST containing 5% nonfat milk. The PVDF membranes were incubated with the primary antibody overnight at 4°C, washed three times with TBST, incubated with a horseradish peroxidase-conjugated secondary antibody for 1 h at room temperature, and then developed with a chemiluminescent reagent (SuperSignal^®^ West Pico Chemiluminescent Substrate, PIERCE). Primary antibodies were rabbit anti-human Phospho-(Ser/Thr) ATM/ATR substrate antibody (Cell Signaling Technology) or rabbit anti-human β-Actin antibody (Cell Signaling Technology).

### Nuclear protein extraction

All nuclear-protein-extraction steps were conducted on ice or at 4°C. Cell pellets were re-suspended in 10 volumes of ice-cold buffer A (10 mM Tris–HCl (pH7.4), 5 mM MgCl_2_, 10 mM NaCl, 1 mM DTT, plus proteinase inhibitor cocktail (Roche), phosphatase inhibitor cocktail (Roche)) and incubated on ice for 20 min. Then ice-cold buffer B (10 mM Tris–HCl (pH7.4), 5 mM MgCl2, 10 mM NaCl, 1 mM DTT, proteinase inhibitor cocktail, phosphatase inhibitor cocktail, 10% NP-40) was added, adjusting the final concentration of NP-40 to 0.5%. After vigorous vortexing on the highest setting for 5 s, the tube was incubated on ice for 1 min, then vortexed for another 5 s. The extract was centrifuged for 5 min at 500 × g, and the supernatant fraction (cytoplasmic extract) removed and saved. The pellet was washed with 5 volumes of ice-cold buffer A to minimize cytoplamic protein contamination, then re-centrifuged for 5 minutes at 500 × g to obtain the nuclei as pellet. The nuclear pellet was resuspended in 10 volumes of freshly prepared buffer C (20 mM HEPES-KOH (pH7.9), 1.5 mM MgCl_2_, 0.5 M NaCl, 1 mM DTT, 0.2 mM EDTA, 20% (V/V) glycerol, proteinase inhibitor cocktail, phosphatase inhibitor cocktail). The nuclear proteins were solubilized by vortexing 3 times for 15 s at 10 min intervals, after which the extract was centrifuged at 16000 × g for 30 min. The supernatant (nuclear proteins extract) was immediately transferred to a pre-chilled tube. The purity of the nuclear and cytoplasmic protein extracts was evaluated by Western Blot for Histone H3 (as a nuclear protein marker) and GAPDH (as a cytoplasmic protein marker) by using rabbit anti-human GAPDH Antibody (Cell Signaling Technology) and rabbit anti-human Histone H3 Antibody (Cell Signaling Technology) as primary antibodies.

### Protein digestion and phosphopeptide enrichment

Proteins were precipitated with 3 volumes 50% acetone/50% ethanol/0.1% acetic acid on ice for 1 h. After centrifugation at 10000 × g for 15 min, the protein pellet was resuspended in 8 M urea/0.2 M Tris, pH 8/4 mM CaCl_2_. Proteins were reduced with 10 mM DTT for 1 h at 56°C, alkylated with 40 mM iodoacetamide for 30 min at room temperature in the dark. After diluting sample 7-fold, trypsin (Worthington) was added in a 1:50 (trypsin/protein) w/w ratio, and incubated overnight at 37°C.

Tryptic peptides from whole cell or nuclear protein extracts were loaded onto a 2 g Sep-Pak C18 column (Waters), washed twice with 10 mL 1% acetic acid, eluted with 7 mL 80% acetonitrile containing 0.1% acetic acid, lyophilized using a speed-vac, resuspended in 400 μL 1% acetic acid and loaded onto a mini column of 40 μL IMAC resin (prepared as previously described [46,47]). The IMAC minicolumn was washed twice with 40 μL wash buffer containing 25% acetonitrile, 100 mM NaCl and 0.1% acetic acid, then washed once each with 40 μL 1% acetic acid and 20 μL deionized water, eluted with 120 μL 6% NH_4_OH, and dried under vacuum. The IMAC column flow-through, which contains mainly non-phosphopeptides, was also collected.

### HILIC workflow

Enriched phosphopeptides and non-phosphopeptides were fractionated separately using a TSKgel Amide-80 column (2.0 mm × 150 mm, 5 μm particle size, 200 Å pore size) (TOSOH Bioscience) on a Agilent 1200 system (Agilent Technologies). A 60 min elution gradient was used for phosphopeptide separation with 90% ACN, 0.005% TFA as Buffer A and 0.005% TFA as Buffer B. The gradient elution profile composed of 0%-12% B for 5 min, 12-30% B for 25 min, 30-90% B for 5 min, then maintained at 90% B for 5 min, followed by 10-100% A for 5 min, ending at 100% A for 15 min. The flow rate was 0.15 mL/min. UV absorbance was monitored at 215 nm. A total of 26 0.3 mL fractions were collected. Fractions were dried via vacuum. Non-phosphopeptides were fractionated in essentially the same manner, except that the gradient elution profile was 0%-5% B for 5 min and then 5-30% B for 25 min.

### RPLC-ESI-MS/MS

A QSTAR ELITE mass spectrometer (Applied Biosystems) was coupled with an online Eksigent nano MDLC systems utilizing a nanospray ionization source. Peptides were first enriched with a CapTrap column (0.5 mm × 2 mm, MICHROM Bioresources, Inc.) followed by elution into an integrated nanoscale analytical column (MAGIC C18AQ, 100 μm × 150 mm, 3 μm particle size, 200 Å pore size, MICHROM Bioresources, Inc.). Mobile phase A (2% ACN, 0.1% formic acid) and mobile phase B (98% ACN, 0.1% formic acid) were used to establish a 130 min gradient comprised of 5 min 5% B, then 25 min 5-15% B, followed by 55 min 15-40% B, then 15 min 40-80% B, maintained at 80% B for 10 min, then 5 min 80-5% B, finally maintained at 5% B for 15 min. The flow rate was ~300 nL/min. We conducted MS from 400 to 1800 amu, with 1 s time spans. For MS/MS analysis, each scan cycle consisted of one full-scan mass spectrum (with m/z ranging from 400 to 1800 and charge states from 2 to 5) followed by five MS/MS events. Threshold count was set to 30, and the exclusion window was 90 s. Mass tolerance was 50 mDa. Automatic Collision Energy and Automatic MS/MS Accumulation were selected.

### Database searching

The raw data collected by QSTAR ELITE were presented by Mascot Daemon (version 2.2.2) (Matrix Science, London, UK) to an in-house MASCOT server (version 2.2) (Matrix Science, London, UK) and Distiller (version 2.2.1.2) (Matrix Science, London, UK). Briefly, peak lists were generated by Distiller and searched against a target/decoy SwissProt human protein database (version 57.7; 20405 sequences) by MASCOT server. Spectra match criteria were as follows: Fixed modification was carbamidomethyl at Cys residue, whereas variable modifications were oxidation at Met residue, and phosphorylation at Ser, Thr or Tyr residues, additionally Arg10 and Lys8 were set as exclusive modifications, which is a useful setting in MASCOT that can be thought of as a choice of fixed modifications to speed up the search and reduce the significance thresholds, and taxonomy was set to “human”. Peptide and MS/MS tolerances were 50 ppm and 0.2 Da, respectively. The peptide charges were 2+, 3+, 4+, or 5+, allowing up to two missed cleavages. The significance threshold was *p* < 0.05.

### Quantitation analysis

The rov data obtained from database search were opened by Mascot Distiller for quantitation. For the quantitation analysis, we set Fraction, Correlation and Std. Err at 0.5, 0.9 and 0.2, respectively. The peptide ratios were calculated as weighted average ratios (ion intensity versus ratio) if several spectra for the same peptide were available. Protein ratios were also calculated as the weighted average ratios (ion intensity versus ratio). In a few situations, outliers were removed for more accurate quantitation. The median of all quantitation data from non-phosphopeptides was used to normalize the peptide ratios.

### STRING network analysis

Identified proteins (accession number) were presented to STRING 8.2 database (http://string-db.org/) [35] to construct networks. Then networks were extracted and loaded into Cytoscape (version 2.6.3) (http://www.cytoscape.org/) [48] for compilation and visualization. Protein networks identified by STRING consisted of protein IDs (nodes) and protein-protein interactions (edges). Only interactions with a score of 0.400, which represents the default medium confidence level in STRING, were kept.

### NetworKIN analysis

In order to predict potential kinases for quantified phosphosites, we used the NetworKIN algorithm which augments motif-based predictions with the network context of kinases and phosphoproteins and can assign a specific kinase to an identified in vivo phosphosite with a 2.5-fold higher accuracy than previous methods such as Scansite and NetphosK [49]. After loading the data to NetworKIN 2.0 (http://networkin.info/version_2_0/newPrediction.php), we obtained sif data which could be rendered with Cytoscape (version 2.6.3). Manual network layout was then performed to display interactions between kinases and phosphosites, using color-coding to indicate fold increase or decrease.

## Competing interests

The authors declare that they have no competing interests.

## Authors’ contributions

XC, GML and LG designed the study. XC performed sample preparation and cell experiments. XC and YZ detected samples by operating LC-MS/MS system. XC analyzed data and wrote the draft manuscript. GML and LG finalized the manuscript. All authors read and approved the final manuscript.

## Supplementary Material

Additional file 1: Table S1 Detail information in proteins identified and quantified in whole cell and nuclear extracts from MNNG-treated TK6 and MT1 cells. ‘L’ (light) represents MNNG-treatment group, and ‘H’ (heavy) represents mock-treatment group. Click here for file

Additional file 2: Table S2 Detail information in phosphopeptides identified and quantified in whole cell and nuclear extracts from MNNG-treated TK6 and MT1 cells. ‘L’ (light) represents MNNG-treatment group, and ‘H’ (heavy) represents mock-treatment group. Click here for file

Additional file 3: Table S3 Detail information in changes respectively in protein expression and protein phosphorylation from MNNG-treated TK6 and MT1 cells. ‘L’ (light) represents MNNG-treatment group, and ‘H’ (heavy) represents mock-treatment group. Click here for file

Additional file 4: Table S4 Detail information in phosphosites quantified in both TK6 and MT1 nuclear extract. ‘L’ (light) represents MNNG-treatment group, and ‘H’ (heavy) represents mock-treatment group. Click here for file

Additional file 5: Figure S1 Examples of peptide tandem mass spectra from differentially-regulated nuclear phosphoproteins from MNNG-treated TK6 and MT1 cells. Displayed were mass spectra of peptide pairs prepared using SILAC method, with the “light” labeling as MNNG-treatment group. Click here for file

Additional file 6: Figure S2 Protein Networks in TK6 nuclear extract. Protein phosphorylation and abundance data in TK6 nuclear extract were analyzed with the STRING database. A GO biological process analysis was performed to extract several representative subgroups (including “RNA processing and splicing”, “Cell cycle” and “Transcription”), as well as a complex network composed of 1285 proteins (nodes) and 8427 connections (edges). If both protein expression and phosphorylation data were available, only information in phosphorylation was shown. Click here for file

Additional file 7: Figure S3 Kinase-substrate interaction networks in TK6 nuclear extract. NetworKIN algorithm was used to predict potential kinases for quantified phosphosites in “Cell cycle” and “RNA processing and splicing” biological processes. The color of the phosphosites represented their alteration after MNNG treatment. Click here for file
