# Supplementary figures and images for: Proteomic analysis of mismatch repair-mediated alkylating agent-induced DNA damage response
Source: Cell Biosci. 2013 Sep 19;3:37. doi: 10.1186/2045-3701-3-37 (PMC3848634; doi:10.1186/2045-3701-3-37)

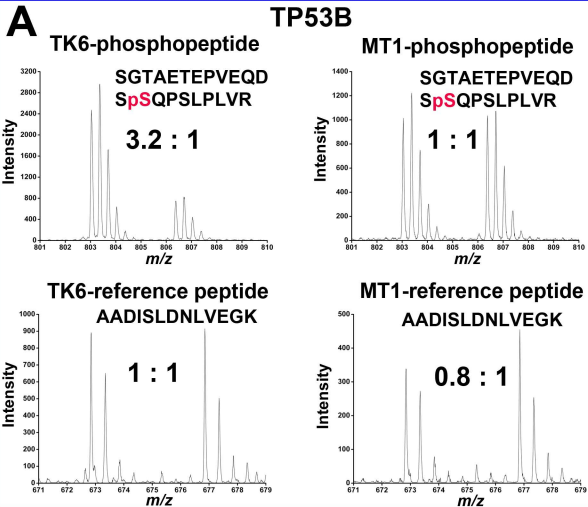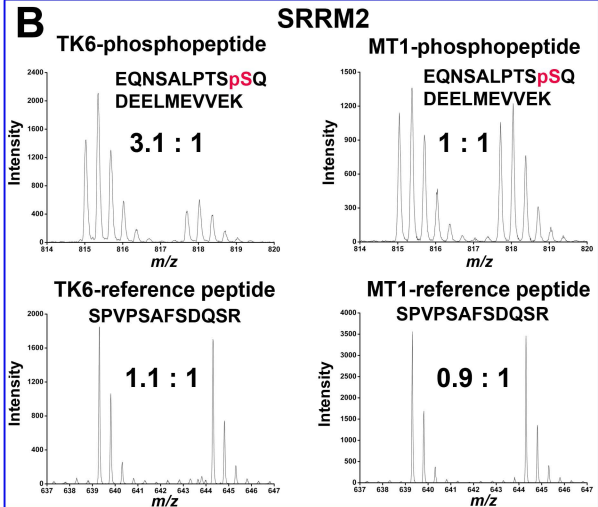

Supplement: Additional file 5: Figure S1 — Examples of peptide tandem mass spectra from differentially-regulated nuclear phosphoproteins from MNNG-treated TK6 and MT1 cells. Displayed were mass spectra of peptide pairs prepared using SILAC method, with the “light” labeling as MNNG-treatment group. [file 2045-3701-3-37-S5.pdf]

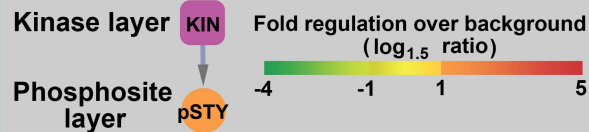

## Cell cycle

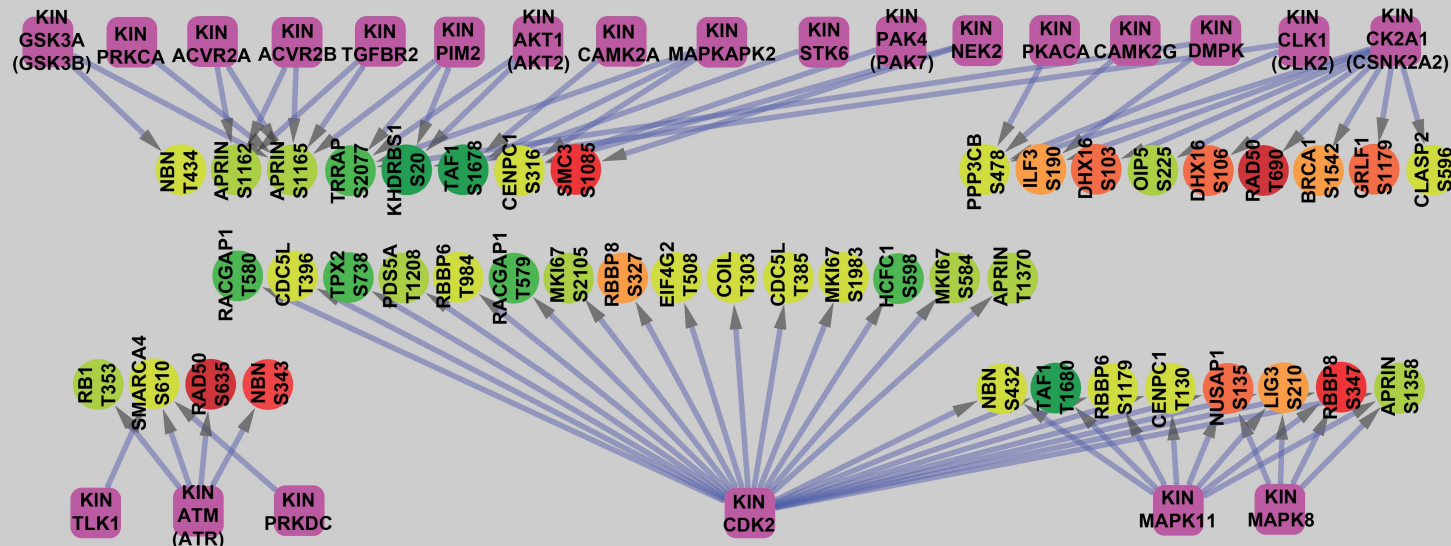

## RNA processing and splicing

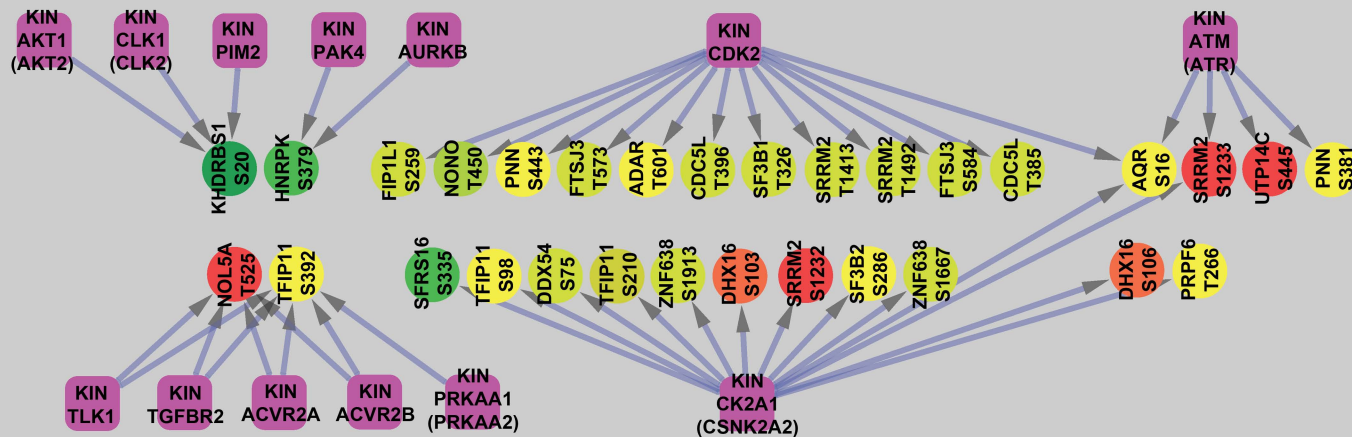

Supplement: Additional file 7: Figure S3 — Kinase-substrate interaction networks in TK6 nuclear extract. NetworKIN algorithm was used to predict potential kinases for quantified phosphosites in “Cell cycle” and “RNA processing and splicing” biological processes. The color of the phosphosites represented their alteration after MNNG treatment. [file 2045-3701-3-37-S7.pdf]
