# Supplementary material for: Proteomic analysis of mismatch repair-mediated alkylating agent-induced DNA damage response
Source: Cell Biosci. 2013 Sep 19;3:37. doi: 10.1186/2045-3701-3-37 (PMC3848634; doi:10.1186/2045-3701-3-37)
Supplement: Additional file 6: Figure S2 — Protein Networks in TK6 nuclear extract. Protein phosphorylation and abundance data in TK6 nuclear extract were analyzed with the STRING database. A GO biological process analysis was performed to extract several representative subgroups (including “RNA processing and splicing”, “Cell cycle” and “Transcription”), as well as a complex network composed of 1285 proteins (nodes) and 8427 connections (edges). If both protein expression and phosphorylation data were available, only information in phosphorylation was shown. [file 2045-3701-3-37-S6.pdf]

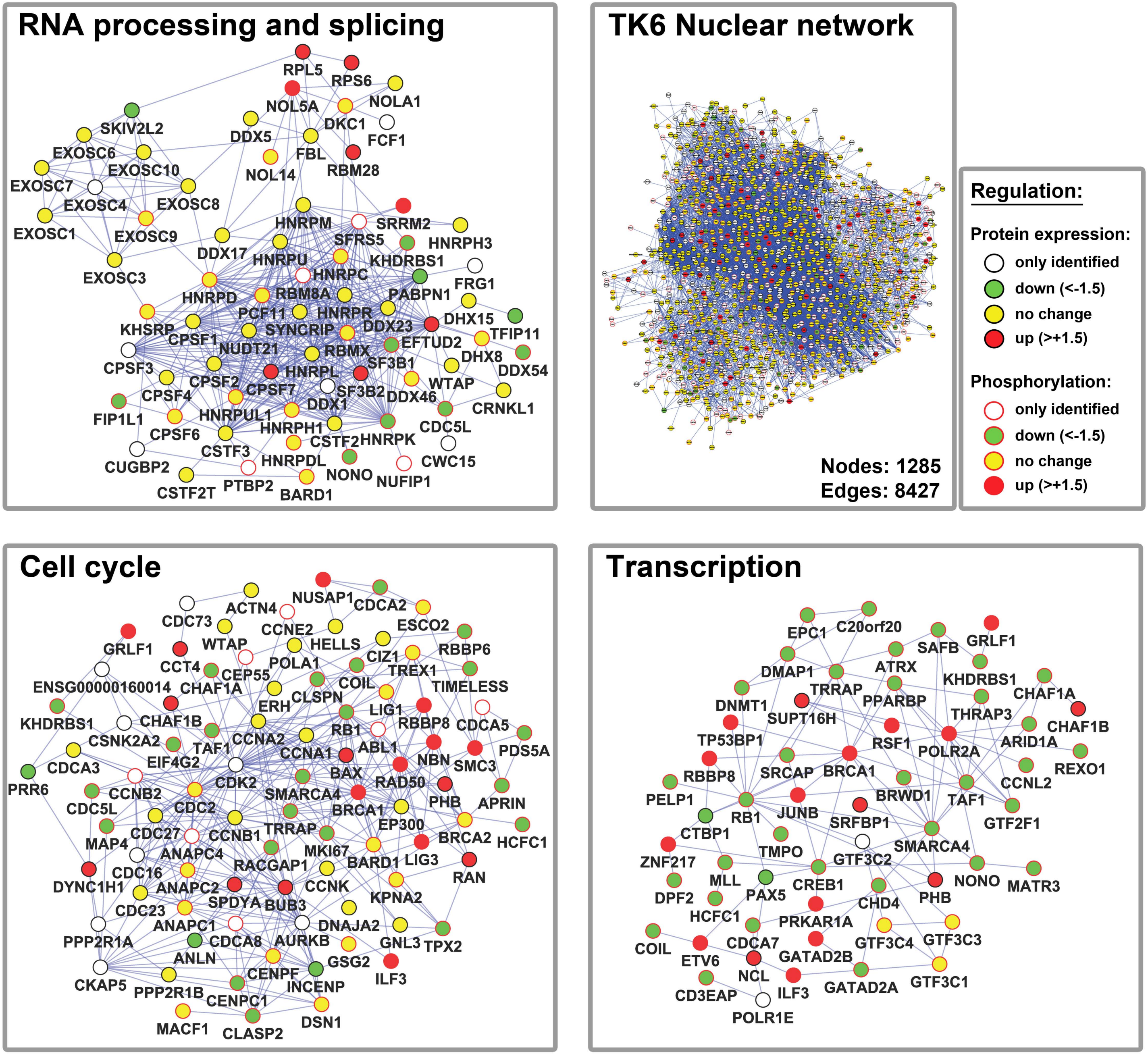

RNA processing and splicing
RPL5
RPS6
NOL5A
NOLA1
DKC1
FCF1
SKIV2L2
DDX5
EXOSC6
FBL
EXOSC10
NOL14
RBM28
EXOSC7
EXOSC4
EXOSC8
EXOSC1
EXOSC9
HNRPM
SRRM2
DDX17
SFRS5
HNRPH3
HNRPU
KHDRBS1
EXOSC3
HNRPC
FRG1
HNRPD
RBM8A
PABPN1
PCF11
HNRPR
DHX15
KHSRP
SYNCRIP
DDX23
TFIP11
CPSF1
EFTUD2
NUDT21
RBMX
DHX8
CPSF3
HNRPL
SF3B1
DDX54
CPSF2
CPSF7
SF3B2
WTAP
CPSF4
DDX1
DDX46
CRNKL1
FIP1L1
HNRPUL1
HNRPH1
CDC5L
CPSF6
CSTF2
HNRPK
CSTF3
HNRPDL
CWC15
CUGBP2
NONO
NUFIP1
CSTF2T
PTBP2
BARD1
TK6 Nuclear network
Nodes: 1285
Edges: 8427
Regulation:
Protein expression:
only identified
down (<-1.5)
no change
up (>+1.5)
Phosphorylation:
only identified
down (<-1.5)
no change
up (>+1.5)
Cell cycle
NUSAP1
CDCA2
ACTN4
CDC73
CCNE2
ESCO2
WTAP
HELLS
GRLF1
RBBP6
CIZ1
CCT4
POLA1
CEP55
TREX1
COIL
ENSG00000160014
CHAF1A
TIMELESS
CLSPN
ERH
LIG1
KHDRBS1
CHAF1B
RBBP8
CDCA5
RB1
CSNK2A2
CCNA2
ABL1
PDS5A
TAF1
CDCA3
CCNA1
NBN
EIF4G2
SMC3
BAX
PRR6
CDK2
RAD50
CCNB2
SMARCA4
PHB
APRIN
CDC5L
CDC2
BRCA1
EP300
MAP4
CDC27
TRRAP
CCNB1
BRCA2
HCFC1
ANAPC4
MKI67
LIG3
CDC16
RACGAP1
BARD1
RAN
DYNC1H1
ANAPC2
CCNK
SPDYA
BUB3
KPNA2
CDC23
ANAPC1
DNAJA2
PPP2R1A
CDCA8
AURKB
GNL3
TPX2
ANLN
GSG2
CENPF
ILF3
CKAP5
INCENP
PPP2R1B
CENPC1
MACF1
CLASP2
DSN1
Transcription
C20orf20
EPC1
GRLF1
SAFB
ATRX
DMAP1
KHDRBS1
TRRAP
CHAF1A
PPARBP
DNMT1
THRAP3
SUPT16H
CHAF1B
TP53BP1
RSF1
ARID1A
POLR2A
REXO1
RBBP8
SRCAP
BRCA1
CCNL2
PELP1
BRWD1
TAF1
JUNB
RB1
GTF2F1
SRFBP1
CTBP1
SMARCA4
TMPO
ZNF217
GTF3C2
NONO
MATR3
MLL
CREB1
PHB
DPF2
PAX5
CHD4
HCFC1
PRKAR1A
GTF3C3
GTF3C4
COIL
CDCA7
GATAD2B
ETV6
NCL
GTF3C1
GATAD2A
CD3EAP
ILF3
POLR1E
